# Chronic Unpredictable Mild Stress Causing Cardiac and Thoracic Spinal Cord Electrophysiological Abnormalities May Be Associated with Increased Cardiac Expression of Serotonin and Growth-Associated Protein-43 in Rats

**DOI:** 10.1155/2018/8697913

**Published:** 2018-03-07

**Authors:** Zhengjiang Liu, Hua Liu, Zhi Huan Zeng

**Affiliations:** ^1^Department of Cardiology, The Six Affiliated Hospital of Guangzhou Medical University, Qingyuan People's Hospital, Qingyuan, China; ^2^Department of Cardiology, The First Affiliated Hospital of Guangdong Pharmaceutical University, Guangzhou, China

## Abstract

**Background:**

The aim of this study was to investigate the potential mechanisms by which chronic unpredictable mild stress (CMS) might induce cardiovascular disease.

**Methods:**

Twenty male Sprague-Dawley rats (weighing 180–250 g) were divided into the CMS group (CMS for 3 weeks) and control group (*n* = 10/group). Sucrose solution consumption, sucrose solution preference rate, and the open field test (horizontal and vertical movements) were used to confirm the establishment of the CMS model. Heart rate was determined in Langendorff-perfused hearts, and field action potential duration (FAPD) was measured in cardiac atrial tissue, cardiac ventricular tissue, and thoracic spinal cord segments 1–5. The expressions of serotonin (5-HT) and growth-associated protein-43 (GAP-43) in cardiac ventricular tissue were analyzed using immunohistochemistry and immunofluorescence.

**Results:**

Compared with the control group, sucrose solution consumption, sucrose solution preference rate, horizontal movement, and vertical movement were significantly lower in the CMS group (*P* < 0.01). The CMS group exhibited significant decreases in atrial and ventricular FAPDs (*P* < 0.05), as well as significant increases in heart rates (*P* < 0.05) and T1–5 spinal cord FAPD (*P* < 0.01), as compared with the control group. The expressions of 5-HT and GAP-43 in cardiac ventricular tissue were significantly higher in the CMS group than in controls (*P* < 0.01).

**Conclusions:**

CMS causes cardiac and T1–5 spinal cord electrophysiological abnormalities as well as increased cardiac expression of 5-HT and GAP-43, indicating that CMS could potentially increase the risk of cardiovascular disease.

## 1. Introduction

The incidence of cardiovascular disease in China has increased progressively in recent years [1]. In addition to the various established risk factors for cardiovascular disease [2], depression is now recognized as being associated with cardiovascular disease [3–7]. A variety of mechanisms have been proposed to underlie the relation between depression and cardiovascular disease [8, 9], but the mechanism by which depression induces cardiovascular disease remains uncharacterized.

The rat model of chronic unpredictable mild stress (CMS) is a widely used animal model of depression [10] because it simulates depression in the face of social environmental stress and thus may mimic the development of depression in humans more closely than other models [11–14]. Previous studies have shown abnormal results in the open field test and decreased sucrose consumption in rodents with experimental depression [15, 16]; hence, these parameters are often used as outcome measures in rodent models of CMS.

One possible link between depression and cardiovascular disease is 5-hydroxytryptamine (5-HT, also known as serotonin). Disturbances in 5-HT signaling have been implicated in the pathogenesis of major depressive disorder [17, 18]. Serotonergic neurons are widely distributed in the nervous system and are thought to regulate the functions of the visceral sensory, motor, and autonomic nervous systems [19]. 5-HT and its receptors are widely distributed in the cardiovascular system, where 5-HT can evoke various acute and chronic harmful effects [20]. Therefore, it could be hypothesized that alterations in 5-HT signaling contribute to the association between depression and cardiovascular disease [21].

Growth-associated protein-43 (GAP-43) is an endogenous neuron growth-dependent protein that plays an important role in neurodevelopment, axonal regeneration, maintenance of synaptic function, and regulation of neurotransmitter release. Several studies have suggested that depression is associated with alterations in GAP-43 expression in the brain, indicating abnormalities in neuronal plasticity, but these studies have been inconsistent in their findings [22–25]. Interestingly, changes in GAP-43 expression in the nucleus of the solitary tract have been proposed to contribute to autonomic dysregulation [26]. Hence, it can be hypothesized that changes in GAP-43 expression and neuronal plasticity are another potential mechanism linking depression and cardiovascular disease. Notably, a previous study found that rats subjected to CMS exhibited abnormalities in their electrocardiogram, including arrhythmias, prolongation of tissue field action potential duration in both cardiac tissue and thoracic T1–5 spinal cord nerves, pathologic changes in the myocardium, and increased expression of GAP-43 in the heart [27].

In the present study, we used CMS rat models and multielectrode array to investigate the electrophysiological changes in T1–5 spinal cord nerves as well as cardiac atrial and ventricular tissues. In addition, we studied the impact of CMS on 5-HT and GAP-43 expression in order to gain insight into the possible mechanisms by which chronic stress-induced depression might lead to the occurrence of cardiovascular disease. 

## 2. Materials and Methods

### 2.1. Animals

A total of 20 specific pathogen-free (SPF) grade male Sprague-Dawley rats (obtained from the Experimental Animal Center of Xinjiang Medical University), weighing 180–250 g, were divided into the control group and CMS group. Rats were housed in a SFP facility at 21 ± 2°C and a humidity of 50 ± 10%, under a 12/12 h light/dark cycle, with free access to food and drinking water, and the animals were allowed to acclimatize to their environment for 1 week before use. The experiments were approved by the Animal Laboratory Administrative Center and the Institutional Ethics Committee of the First Affiliated Hospital of Xinjiang Medical University (20080317002) and performed in accordance with the National Institutes of Health Guidelines.

### 2.2. Preliminary Work and General Procedure

**Figure 1:**
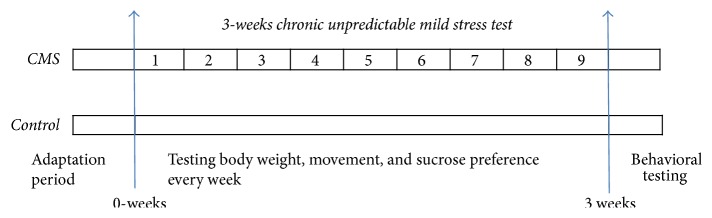
General procedure in this study. The nine types of environmental stressors for the CMS group. (1) Behavior restrictions; (2) overnight lighting; (3) tilted cage for 24 h; (4) clustered feeding; (5) ice water swimming; (6) day and night reversal; (7) soiled cage; (8) wet cage; and (9) noise stimulation. The stressful stimulus was given at 8:00 a.m. every morning, and the stimuli were applied in a random order to ensure the animals could not predict the stimulus to be used. A total of nine types of stimuli were applied, and the stimulus type was randomly assigned each day. The control group of animals was housed for the same period of time (three weeks) without exposure to stressful stimuli. *n* = 10/group.

All rats were allowed to adapt to their new environment within two weeks of their arrival. Prior to the experiments, 60 rats with a horizontal score between 30 and 50 in the out-of-box exercise test were selected. Qualified animals were randomly divided into the two groups. One group was housed individually in separate cages and subjected to 3-week unpredictable mild stress as CMS group (*n* = 10). The other group was placed in standard conditions with food and water cages without any stress as control (*n* = 10) for 3 weeks (Figure 1). All rats were housed in a SFP facility at 21 ± 2°C and a humidity of 50 ± 10% collective cages, under a 12/12 h light/dark cycle, with free access to food and drinking water.

### 2.3. CMS Model

The CMS model was established according to previous studies [28, 29]. The social stressors used for rats in the CMS group were isolation (i.e., the animals were kept separately) and social stress (the animals were placed in the empty cage of another male). The environmental stressors used were as follows: (1) behavioral restriction in small cages (with breathing holes); (2) continuous lighting overnight; (3) tilting of the cage to an angle of 40° (along the vertical axis) for 24 h; (4) feeding in clusters; (5) swimming in cold (4°C) water for 5 min; (6) reversal of the light/dark cycle for 24 h; (7) dirty cage for 24 h; (8) wet litter for 24 h (a mixture of 200 mL of water and 100 g of sawdust); and (9) noise stimulation (60 Hz) for 1 h. The stressful stimulus was given at 8:00 a.m. every morning, and the stimuli were applied in a random order to ensure that the animals could not predict the stimulus to be used. A total of nine types of stimuli were applied, and the stimulus type was randomly assigned each day. The control group of animals was housed for the same period of time (three weeks) without exposure to stressful stimuli.

The core feature of depression is a lack of pleasure, which manifests in rats as a reduction in sucrose preference. The sucrose preference test was performed using the method described by D'Aquila and colleagues [30–32]. Forty-eight hour before experiment, the rats were trained to the sucrose solution (1%) test: that is, they had sucrose solution for a whole day. On the other day, they were given sucrose solution and water for 24 h. The adaptation period lasted for a total of 48 h. The rats were fasted on the 3rd day. After 24 h of fasting, plastic bottles containing preweighed water and 1% sucrose solution were placed in the cages, and the rats were allowed to take liquid for 1 h. Then the bottles were taken out and weighed to determine the liquid consumption amount (g). It was found that stable results could be obtained by using 1% sucrose solution and water after a 24 h fast, while unstable and biased results would be obtained using a shorter fast (data not shown). The preference rate for sucrose solution (%) was calculated as sucrose solution consumption/(sucrose solution consumption + water consumption) × 100%.

### 2.4. Open Field Test

The open field test is used as a measure of the spontaneous activity of each rat and was conducted as previously described [33]. The conditions were dark and quiet room with a visibility of 5 m. An uncovered case (of length 100 cm, width 50 cm, and height 100 cm) was used as the experimental device. The bottom of the case was painted black and marked with white lines to create squares of 20 cm × 20 cm. At the beginning of the test, the rat was placed in the central square, and the following activities were observed for 300 s: horizontal movement, measured as the number of squares passed (passing of a square was defined as all feet within the same square), and vertical movement, defined as the total number of instances where the rat was upright on its hind legs, indicating the rising of its two front paws or the climbing of a wall. This test was performed before and after establishment of the CMS model.

### 2.5. Electrophysiological Recordings

Electrophysiological recordings were performed as previously described [34, 35]. Each rat was weighed, anesthetized with chloral hydrate (0.3 mL/100 g, i.p.), and fixed in the supine position. The thoracic cavity was opened, and the heart was excised and perfused by the Langendorff technique [36] at 1.5 mL/min for 30 min. Then, self-made copper microelectrodes were attached to the atrial and ventricular surfaces to enable recording of field action potentials from the atrial and ventricular tissues using a 16–128 cardiac electrophysiology recorder (Jinjiang Electronic Science and Technology Co., Ltd., Sichuan, China). During recording, the adsorption electrodes were attached perpendicularly to the atria and epicardium, and a negative pressure was applied to firmly attach the electrodes to the epicardial surface of the heart. The atrial single-phase action potential duration was 50–100 ms and the frequency bandwidth was 0.02–1000 Hz. The ventricular single-phase action potential duration was 50–100 ms, frequency bandwidth was 0.006–10,000 Hz, and sampling rate was 10K.

The application of multielectrode array has become increasingly common in neurology and cardiology [37, 38] and can achieve a high spatial and temporal resolution in stimulation and recordings in vitro (cell and tissue culture) [39]. Since multielectrode array records the extracellular field potential, it allows nondestructive measurements at the cellular level [40]. The microelectrode array recording system included 8 × 8 TiN electrodes on a microsized glass surface with a diameter of 5 mm, with a minimum electrode diameter of 10 *μ*m and a minimum electrode spacing of 30 *μ*m. The in vitro tissues, cells, or slices are directly and tightly placed on the microelectrode array. It can record 60 sites of extracellular field potential synchronously. The T1–5 section of the spinal cord was excised from the rat and placed in cold Krebs solution (119 mmol/L NaCl, 4.7 mmol/L KCl, 2.5 mmol/L CaCl_2_, 1.2 mmol/L KH_2_PO_4_, 1.2 mmol/L MgSO_4_, 25 mmol/L NaHCO_3_, and 1.1 g/L sucrose, pH 7.36). The T1–5 spinal cord section was placed in a multielectrode array, and field action potentials were recorded at 500 mV and 1000 mV. The signals obtained from 60 channels were collected by a MEA1060 amplifier and analyzed using MC-cardb software (Multichannel Systems, Reutlingen, Germany). Field action potential durations were determined using the MC_Rack software (Multichannel Systems).

### 2.6. Immunohistochemistry to Detect 5-HT in Tissue Sections from the Left Ventricle

Immunohistochemistry for 5-HT was performed as previously described [41]. After field action potentials had been recorded, the heart was fixed and embedded in paraffin, and 0.5 cm^3^ tissue blocks were cut vertically along the left ventricular long axis from the apex of the heart. A 5 *μ*m section was cut from each paraffin block of heart tissue. All sections were incubated at 60°C for 60 min, deparaffinized in xylene, rehydrated, and incubated with fresh 0.03% H_2_O_2_ in 100% methanol for 10 min at room temperature to block endogenous peroxidase activity. Microwave antigen retrieval was performed in 0.01 M citrate buffer (pH 6.0) in a 700–800 W microwave oven. After rehydration using a graded ethanol series, the sections were heated in water to 95°C for 15 min. Nonspecific binding sites were blocked by incubation with 2% bovine serum albumin for 60 min. Anti-5-HT primary antibody (1 : 100; PB0442, Wuhan Boster Biological Engineering Company, Wuhan, China) was applied for 24 h at 4°C, and horseradish peroxidase-labeled anti-rabbit IgG secondary antibody (rabbit polyclonal Ig antibody SV-002, Wuhan Boster Biological Engineering Co., Ltd.) was applied at 37°C for 30 min. After three washes in phosphate-buffered saline (PBS), the chromogen 3,3′-diaminobenzidine tetrahydrochloride (DAB) was applied as a 0.02% solution containing 0.005% H_2_O_2_ in 50 mmol/L ammonium acetate-citric acid buffer (pH 6.0). The sections were lightly counterstained with Mayer's hematoxylin and mounted.

Three sections from each tissue were observed using an Olympus optical microscope (Olympus, Japan). Five fields (×200) were randomly selected in each section using the Kontron IBAS computerized image analysis system (Carl Zeiss AG, Oberkochen, Germany). The positive reaction area and positive intensity value in the same area were measured, and the positive index (PI) was calculated according to the following formula: PI = (positive reaction area × positive intensity value)/measurement area.

### 2.7. Immunohistochemistry to Detect GAP-43 in Tissue Sections from the Left Ventricle

Immunohistochemistry for GAP-43 was performed as previously described [42]. After field action potentials had been recorded, left ventricular apical tissue was removed, immediately frozen at −20°C using a CM3050 S cryostat (Leica Biosystems, Wetzlar, Germany), and cut into 5 *μ*m sections that were stored at −20°C. Tissue sections were fixed overnight in 4% paraformaldehyde prior to processing. The tissues were blocked in 10% goat serum (Wuhan Boster Biological Engineering Co., Ltd.) for 1 h at room temperature prior to incubation with anti-GAP-43 primary antibody (1 : 100; Wuhan Boster Biological Engineering Co., Ltd.) overnight at 4°C. After three washes in PBS, the sections were incubated with fluorescein isothiocyanate-conjugated goat anti-rabbit IgG secondary antibody (1 : 250 in PBS with 1.5% normal blocking serum) at room temperature for 1 h. The sections were rinsed three times with PBS, mounted with mounting medium, and counterstained with DAPI (4′,6-diamidino-2-phenylindole) to visualize the nuclei. All images were viewed using a MicroTime200 scanning laser confocal microscope (PicoQuant, Berlin, Germany) to visualize the distribution of GAP-43. Five randomly selected fields from each section of the left ventricular apex were analyzed (×200), and the average number of fiber bands stained in each field was taken to represent sympathetic nerve density.

### 2.8. Statistical Analysis

SPSS 16.0 statistical software (SPSS Inc., Chicago, IL, USA) was used for data analysis. All data in the figures are presented as mean ± SD. One-way repeated measures ANOVA was performed to study the changes in body weight and sucrose pre/poststress. One-way ANOVA with environment (control versus CUMS) as the grouping factor was used to compare the groups. If there is a difference, further conduct pairwise comparisons. Use Levene test to test variance homogeneity. If variances are homogeneous, use Tukey method; otherwise use Tamhane's T2. Statistical significance was set at *P* < 0.05.

## 3. Results

### 3.1. Body Weight and Stomach Content of Food

**Figure 2:**
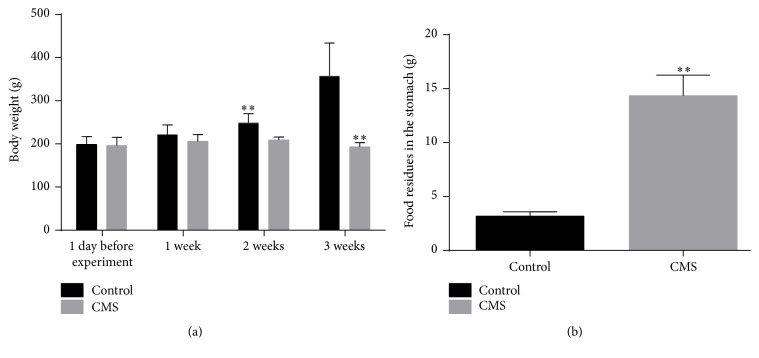
Weight and residual food content in the stomach. (a) The weight of the animals of the two groups was similar at baseline and 1 week, but the CMS group showed no weight gain at 2 and 3 weeks, while the control group gained body weight (*P* < 0.01). (b) The CMS group showed higher gastric food residues than the control group (*P* < 0.01). *n* = 10/group. ^*∗∗*^*P* < 0.01 compared with control group.

Before the start of the experiment and on the 7th day, the rats in the two groups had normal diet and rich and lustrous body hair. The weight of the rats in the control group (baseline: 199 ± 18 g; 7th day: 222 ± 22 g) and CMS group (baseline: 196 ± 19 g; 7th day: 206 ± 16 g) was not significantly different (*P* = 0.70 and *P* = 0.073). After CMS, dirtiness, hair loss, and laziness were observed in the rats. After 14 and 21 days of CMS, the body weight of the rats in the CMS group (14th day: 209 ± 7 g; 21st day: 193 ± 10 g) was significantly lower than that of the control group (14th day: 249 ± 21; 21st day: 358 ± 76 g) (*P* < 0.001 and *P* < 0.001). Rats receiving CMS started to reduce their food intake and voluntary activity and displayed gradually enlarged abdomen and mental exhaustion. The stomach food was weighed at 9:00 a.m. on the day of the experiment, and there was a significant difference (*P* < 0.001) in the gastric food residues in the control group (3.17 ± 0.41 g) compared with the CMS group (14.34 ± 1.92 g) (Figure 2).

### 3.2. Successful Establishment of the CMS Model

#### 3.2.1. Sucrose Preference Test

**Figure 3:**
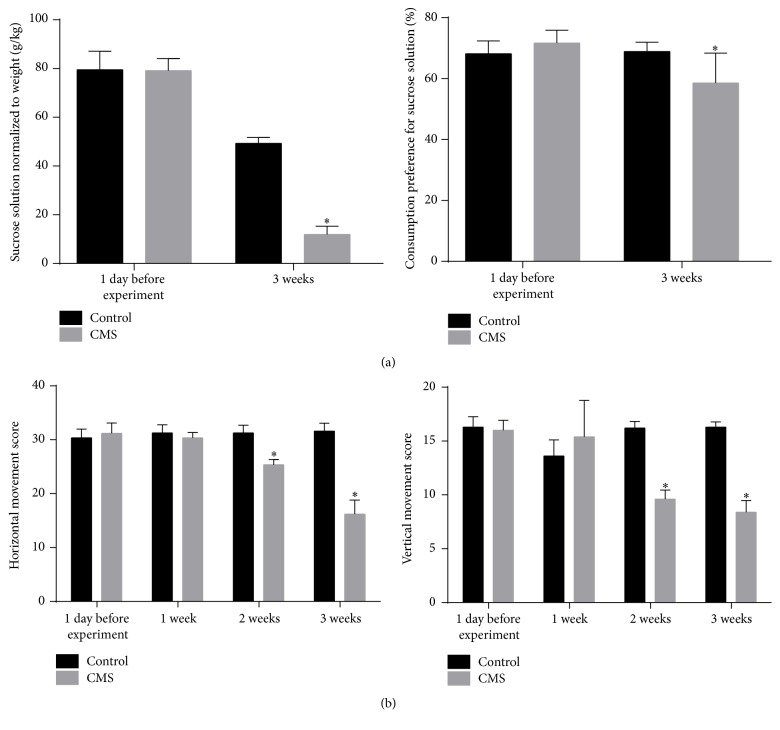
Indicators of stress in rats. (a) Consumption of sucrose solution normalized to body weight and preference for the consumption of sucrose solution. (b) Horizontal and vertical movement score during the open field test. ^*∗*^*P* < 0.01. *n* = 10/group.

There were no significant differences between groups in sucrose solution consumption (normalized to body weight) or the preference rate for sucrose solution before the experiment (Figure 3(a)). By contrast, sucrose solution consumption and preference rate for sucrose solution were significantly lower in the CMS group after three weeks of chronic stress than in the control group (*P* < 0.01; Figure 3(a)), indicating that the CMS group exhibited symptoms consistent with depression and anhedonia. In the open field test, there were no significant differences between groups in horizontal movement and vertical movement scores before the experiment (Figure 3(b)). After three weeks of chronic stress, the CMS group had significantly lower horizontal movement and vertical movement scores than the control group (*P* < 0.01; Figure 3(b)).

### 3.3. Heart Rate, Cardiac Atrial Field Action Potential Duration, Cardiac Ventricular FAPD, and T1–5 Spinal Cord

#### 3.3.1. Field Action Potential Duration Was Significantly Altered in Rats Subjected to CMS

**Figure 4:**
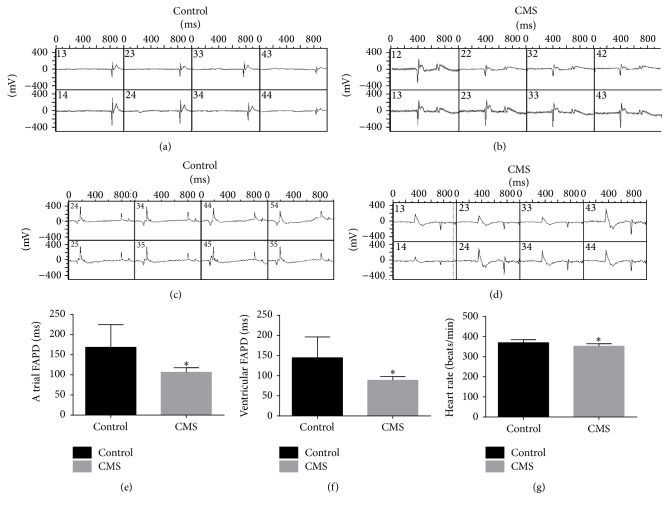
Representative examples of field action potentials recorded from localized regions of cardiac atrial and ventricular tissue. (a) Atrial field action potentials in the control group. (b) Atrial field action potentials in the CMS group. (c) Ventricular field action potentials in the control group. (d) Ventricular field action potentials in the CMS group. (e) Quantification of atrial field action potentials. (f) Quantification of ventricular field action potentials. (g) Quantification of heart rates. ^*∗*^*P* < 0.01. *n* = 10/group.

**Figure 5:**
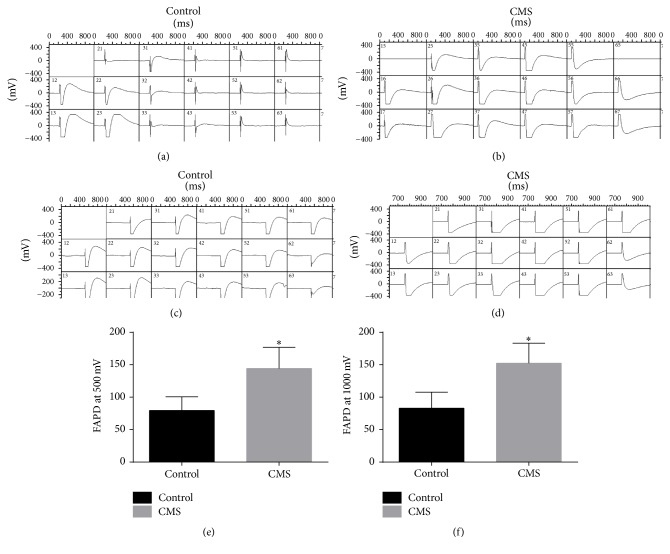
Representative examples of field action potentials recorded from T1–5 spinal cord. (a) T1–5 spinal cord field action potentials (500 mV stimulation) in the control group. (b) T1–5 spinal cord field action potentials (500 mV stimulation) in the CMS group. (c) T1–5 spinal cord field action potentials (1000 mV stimulation) in the control group. (d) T1–5 spinal cord field action potentials (1000 mV stimulation) in the CMS group. (e) Quantification of the T1–5 spinal cord field action potentials at 500 mV stimulation. (f) Quantification of the T1–5 spinal cord field action potentials at 1000 mV stimulation. ^*∗*^*P* < 0.01. *n* = 10/group.

After three weeks of chronic stress, the CMS group exhibited significant decreases in atrial field action potential duration and ventricular field action potential duration compared with the control group (*P* < 0.05; Figures 4(a)–4(f)) as well as a significant decrease in heart rate compared with the control group (*P* < 0.05; Figure 4(g)). The T1–5 spinal cord field action potential duration (at both 500 mV and 1000 mV of stimulation) was also significantly prolonged in the CMS group after three weeks of chronic stress, as compared with the control group (*P* < 0.01; Figure 5).

#### 3.3.2. 5-HT Expression Was Increased in the Cardiac Ventricular Tissue of Rats Subjected to CMS

**Figure 6:**
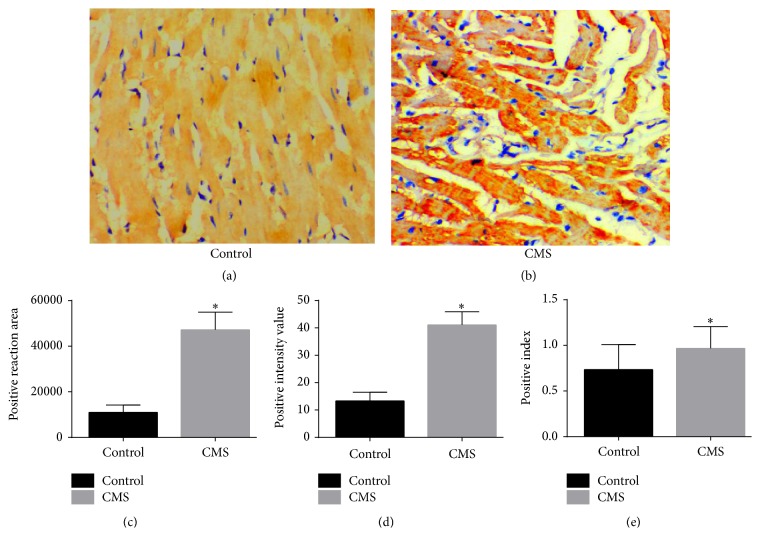
Expression of 5-hydroxytryptamine (5-HT) in cardiac ventricular tissue. (a-b) Representative examples, obtained using immunohistochemistry techniques to stain for 5-HT, are shown. (a) Control group. (b) CMS group. Magnification: ×200. (c) Quantification of the positive reaction area. (d) Quantification of the positive intensity value. (e) Quantification of the positive index. ^*∗*^*P* < 0.01. *n* = 10/group.

5-HT-immunoreactive fibers were evident in the ventricular myocardium of rats from both groups. Myocardial fibers from rats in the control group were neatly arranged without the presence of large quantities of intercellular tissue, and 5-HT-positive nerve fibers projected along the direction of muscle bundles (Figure 6(a)). Compared with controls, myocardial fibers from rats in the CMS group were more dispersed and with larger quantities of intercellular tissue (Figure 6(b)). When the data were quantified, the positive reaction area, positive intensity value, and PI were all significantly higher in the CMS group than in the control group (*P* < 0.01; Figures 6(c), 6(d), and 6(e)).

#### 3.3.3. GAP-43 Expression Was Increased in the Cardiac Ventricular Tissue of Rats Subjected to CMS

**Figure 7:**
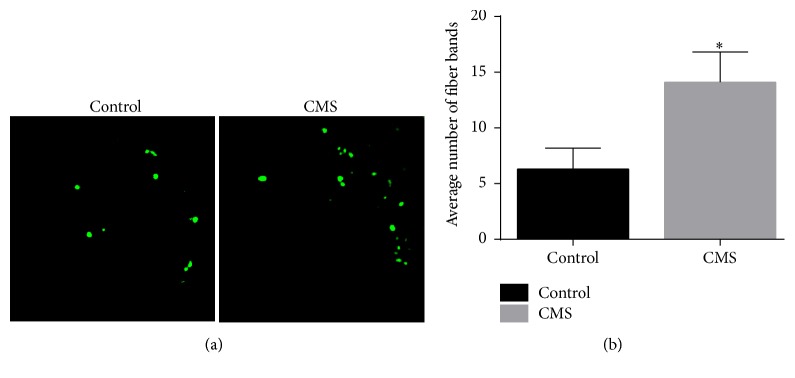
GAP-43 expression in cardiac ventricular tissue. (a) Representative examples, obtained using immunohistochemistry techniques to stain for GAP-43, are shown. Left: control group. Right: CMS group. Magnification: ×200. (b) Quantification of the fiber bands stained. ^*∗*^*P* < 0.01 compared with control group.

GAP-43 expression in cardiac ventricular tissue was significantly higher in the CMS group than in the control group (average number of fiber bands stained: 14.10 ± 2.73 versus 6.30 ± 1.89, *P* < 0.01; Figure 7). Moreover, the morphology of GAP-43 expression was more irregular in the CMS group than in the control group.

## 4. Discussion

The present study showed that rats subjected to CMS had decreased atrial and ventricular field action potential durations, increased heart rate, and prolonged T1–5 spinal cord field action potential duration. Furthermore, the expressions of 5-HT and GAP-43 in cardiac ventricular tissue were higher in the CMS group than in the control group. To the best of our knowledge, this is the first report to show that CMS in rats causes cardiac electrophysiological abnormalities that may be associated with upregulation of cardiac 5-HT and GAP-43.

Rodent models of CMS have been widely used for research into the pathogenesis and treatment of depression [10]. Animals subjected to CMS exhibit an array of symptoms consistent with the development of depression and anhedonia [11–14]. The present study observed that rats subjected to CMS showed decreased sucrose consumption/preference and reduced activity in the open field test, which is consistent with previous research [15, 16], suggesting that CMS model was successfully generated.

The central nerve pathways for 5-HT originate in the midbrain, and some serotonergic fibers project from the medulla oblongata to the spinal cord [43]. In the dorsal raphe nucleus of the midbrain, 5-HT neurons regulate cognition, emotion, and awakening, while in the raphe of the medulla oblongata, 5-HT neurons regulate respiratory and cardiac functions [44, 45]. 5-HT can regulate the structure and function of the heart through 5-HT2B receptors, while an increase in the serum level of 5-HT can cause sinus tachycardia through the excitation of atrial 5-HT4 receptors. Changes in serum 5-HT can induce atrial arrhythmias and even atrial fibrillation [46, 47]. Furthermore, enhanced levels of 5-HT in the blood can cause vasoconstriction, leading to an increase in blood pressure as well as heart valve damage and pulmonary hypertension [48–50]. Multielectrode array recordings revealed that the CMS group had a higher heart rate and showed significant changes in the field action potential durations of the T1–5 spinal cord, cardiac atrial tissue, and cardiac ventricular tissue. Observations of a higher heart rate and shorter cardiac field action potential durations in rats subjected to CMS are in agreement with a previous study [27] and would be consistent with increased cardiac sympathetic activity relative to parasympathetic activity, as has been previously reported [51–53]. Furthermore, it has been suggested that depression is associated with impaired control of cardiac autonomic activity by serotonergic neurons [54, 55]. Interestingly, chronic mental stress can induce asymmetry in midbrain activity and abnormal sympathetic nerve output, increasing the risk of arrhythmia [56, 57]. A novel finding of the present study was that 5-HT expression was increased in the heart. This may be an additional potentially proarrhythmic mechanism associated with depression, since 5-HT is known to promote atrial arrhythmias [47, 58].

An additional observation made in this study was that cardiac expression of GAP-43, a marker of neuronal growth [59], was increased. Depression is associated with dysfunction of 5-HT transmission in the central nervous system [60] and, as described above, alterations in autonomic nervous system function, including increased sympathetic nerve tone. These changes may lead to neuronal remodeling and increased synaptic density, as evidenced by upregulated expression of GAP-43, which may in turn contribute to an augmented sympathetic tone as well as the enhanced levels of 5-HT observed in this study. These changes may represent a long-term adaptive process that leads to dysregulation of the function of organs such as the heart.

There are many possible explanations for stress arrhythmia. In the present study, based on the limited experimental results, we presumed that chronic stress-induced depression could lead to sympathetic balance disorders and increased serotonin neurons, which led to the destruction of the ventricular electrophysiological consistency, and promoted arrhythmias. It is important to underline that this is only one possible mechanism of depression causing arrhythmia. Nevertheless, the abnormalities in the number and distribution of cardiac serotonin neurons and their interactions with the autonomic nervous system, the roles of 5-HT nerve fiber/neurons, and each subtype receptor remain to be further explored. Furthermore, in this study, the changes of resting electrocardiogram before and after experiment in the two groups of living rats during chronic stress were not discussed. Only the change of resting heart rate after the experiment was recorded, and no change of heart rate was dynamically recorded by ECG. Otherwise, the results could further favor our experimental results that autonomic nervous dysfunction could cause electrophysiological changes in the heart under the effect of serotonergic neurons after stress. This point requires further exploration. Another limitation of the study was that the whole-cell patch-clamp recording technique was not used to compare the differences in ion-current in T1–5 spinal cord and atrium and ventricular myocytes between the two groups under the effect of serotonergic neurons after stress. This technique would allow investigating the membrane ion-flux for the changes of field potential duration in the spinal cord and heart in the two groups. In addition, this is an animal experiment to explore stress arrhythmia, and the results still need to be supplemented by more experiments.

## 5. Conclusions

In summary, CMS can lead to abnormalities of cardiac electrophysiology, possibly related to autonomic dysfunction, that potentially increase the risk of arrhythmia. Neuronal remodeling (e.g., of sympathetic nerves) and enhanced levels of 5-HT in the heart may contribute to these mechanisms. Nevertheless, further studies are needed to investigate CMS-induced alterations in the number and distribution of cardiac 5-HT neurons and their relationship with the autonomic nervous system.
